# Nanoparticle-Delivered Transforming Growth Factor-β1 siRNA Induces PD-1 against Gastric Cancer by Transforming the Phenotype of the Tumor Immune Microenvironment

**DOI:** 10.3390/ph15121487

**Published:** 2022-11-29

**Authors:** Fenglei Wu, Xiujuan Xu, Wei Li, Yidong Hong, Huan Lai, Jingzhou Zhang, Xueyu Wu, Kangjie Zhou, Nan Hu

**Affiliations:** 1 Department of Oncology, The First Affiliated Hospital of Kangda College of Nanjing Medical University (The Affiliated Lianyungang Hospital of Xuzhou Medical University), Xuzhou 221004, China; 2 Department of Radiation Oncology, Lianyungang Second People’s Hospital (Lianyungang Cancer Hospital), Lianyungang 222023, China; 3 Center of Research Laboratory, The First Affiliated Hospital of Kangda College of Nanjing Medical University (The Affiliated Lianyungang Hospital of Xuzhou Medical University), Xuzhou 221004, China; 4 Department of Oncology, The Affiliated Lianyungang Hospital of Xuzhou Medical University (The First People’s Hospital of Lianyungang), Lianyungang 222002, China

**Keywords:** nanoparticle, gastric cancer, tumor microenvironment, immunotherapy

## Abstract

Immune checkpoint blockade (ICB) is currently considered to be an important therapeutic method, which obtained FDA approval for clinical use in gastric cancer in 2017. As a new mechanism, it was found that the effect of αPDL1 could be improved by blocking the TGF-β1 signaling pathway, which converts the tumor immune microenvironment from the “immune-excluded phenotype” to the “immune-inflamed phenotype”. Based on this phenomenon, this project was designed to prepare TGF-β1-siRNA-loaded PEG-PCL nanoparticles conjugated to αPDL1 (siTGF-β1-αPDL1-PEG-PCL) since we have linked similar antibodies to PEG-PCL previously. Therefore, MFC tumor-engrafted mice were established to simulate the biological characteristics of converting the phenotype of the immune microenvironment, and to study the anti-tumor effect and possible molecular mechanism. In this study, αPDL1 antibody conjugates markedly increased the cell uptake of NPs. The produced αPDL1-PEG-PCL NPs efficiently reduced the amounts of TGF-β1 mRNA in MFC cells, converting the immune microenvironment of MFC tumors engrafted mice from the “immune-excluded phenotype” to the “immune-inflamed phenotype”. PDL1-harboring gastric cancer had increased susceptibility to αPDL1. The value of this drug-controlled release system targeting the tumor microenvironment in immune checkpoint therapy of gastric cancer would provide a scientific basis for clinically applying nucleic acid drugs.

## 1. Introduction

Gastric cancer (GC) represents a common malignancy in China, with about 400,000 new patients reported each year [1]. Most GC cases are diagnosed at an advanced stage, and despite significant progress in radiotherapeutic and chemotherapeutic treatments, patient prognosis in advanced GC remains poor, and treatment options are limited [2]. Fluoropyrimidine and platinum-containing chemotherapeutics are used in combination with trastuzumab in HER2-positive GC cases as first-line regimen, while taxanes administered with or without ramucirumab as second-line regimen are routinely used for advanced GC (AGC) treatment [3]; however, such treatments do not remarkably improve prognosis, and median survival is approximately one year [4,5]. Most human tumors have abnormal expression of oncogenes or tumor suppressor genes specific to cancer cells whose products are recognized by T cells, which has become a hotspot in tumor immunotherapy research [6]. However, the immunity induced by tumor-associated antigens gradually enters the “exhaustion” phase because of the immunoregulatory factor programmed death receptor 1 (PD1) [7], a CD28 family member that acts as a negative costimulatory receptor on activated T lymphocytes [8]. Therefore, blocking the PD1/PDL1 pathway by anti-PD1 (αPD1) or anti-PD1 ligand 1 (αPDL1) antibodies can potentially revert exhaustion in cytotoxic T cells, preventing the immune escape of malignant cells [8]. Recently, this immune checkpoint blockade (ICB) therapy with monoclonal antibodies has been successfully applied in multiple cancers [9,10,11].

Transforming growth factor β (TGF-β) represents an important protein exerting multiple cell functions in many tissues. TGF-β plays a dual role in tumor development, inhibiting cancer cells in the early stage, but promoting cancer progression in the advanced stage by suppressing immune responses, inducing angiogenic and metastatic pathways, enhancing epithelial-to-mesenchymal transition (EMT), activating fibroblasts and promoting desmoplasia [12]. TGF-β reduces survival in several malignancies [13,14]. There are three TGF-β isoforms in mammalian organisms, including TGF-β1-3, with TGF-β1 being the most abundant [15].

Antitumor immunity in humans comprises three major phenotypes, i.e., the immune-desert, immune-excluded and inflamed phenotypes, all of which have specific mechanisms for preventing immune clearance of the cancer by the host. Tumors with an immune-desert phenotype could result from immunological ignorance, tolerance induction or inadequate T cell priming or activation. Those with immune-excluded phenotype might indicate specific chemokine expression state, vascular factors, or stromal-related suppression. Inflamed tumors are infiltrated by several immune cell types such as immune-suppressive regulatory T cells, myeloid-derived suppressor cells, suppressor B cells and cancer-associated fibroblasts [16]. Malignant tumors with high TGF-β1 expression are mostly of immune-excluded phenotype [17]. In the tumor microenvironment, TGF-β1 activates the matrix to form fibroblasts and deposit collagen in the cell matrix. TGF-β1 participates in the regulation of cytotoxic T lymphocyte (CTL) infiltration into the tumor immune microenvironment of immune-excluded tumors [17]. Sanjeev et al. found that TGF-β1 can reshape the tumor microenvironment and limit the movement of T cells; the combination of TGF-β1 blockers and PDL1 blockers could inhibit the TGF-β1 pathway in stromal cells, therefore facilitating T cell entry in the tumor, stimulating antitumor immunity and causing cancer cell death [17,18]. Moreover, in a mouse model recapitulating the immune-excluded phenotype, these authors demonstrated combined treatment with TGF-β1-blockers and anti- programmed death-ligand 1 (PD-L1) antibodies suppresses the TGF-β1 pathway in stromal cells and promotes T cell infiltration into tumors, converting the immune-excluded phenotype to the inflamed phenotype, with the overall result of potent antitumor immunity and tumor shrinkage [17].

Nanocarriers are frequently used for enhancing drug delivery to the TME; the particles enter through the leaky tumor vasculature, which is referred to as the enhanced permeation and retention (EPR) effect. NP loading with target-specific ligands or antibodies increases the treatment effects by promoting targeted delivery to the tumor microenvironment [19,20,21]. We previously explored strategies for conjugating trastuzumab on the surface of NPs to delay the degradation of nucleic acid drugs [22]. In this study, αPDL1 were linked to poly(ethylene glycol) (PEG) and poly(ε-caprolactone) (PCL) copolymers to achieve αPD-L1-PEG-PCL (Figure 7). TGF-β1 siRNA was encapsulated in αPDL1-PEG-PCL by the double-emulsion solvent evaporation method. Receptor-mediated targeting was achieved by PDL1 overexpression. We hypothesized that αPDL1 can be used not only to target nanoparticles with TGF-β1 siRNA to given cells, converting the immune-excluded phenotype to the inflamed phenotype, but also to convey immune checkpoint blockade, further reversing T cell exhaustion and enhancing the anti-tumor effect of ICB therapy.

## 2. Results

### 2.1. Synthesis of the αPDL1-PEG-PCL Copolymer

**Figure 1:**
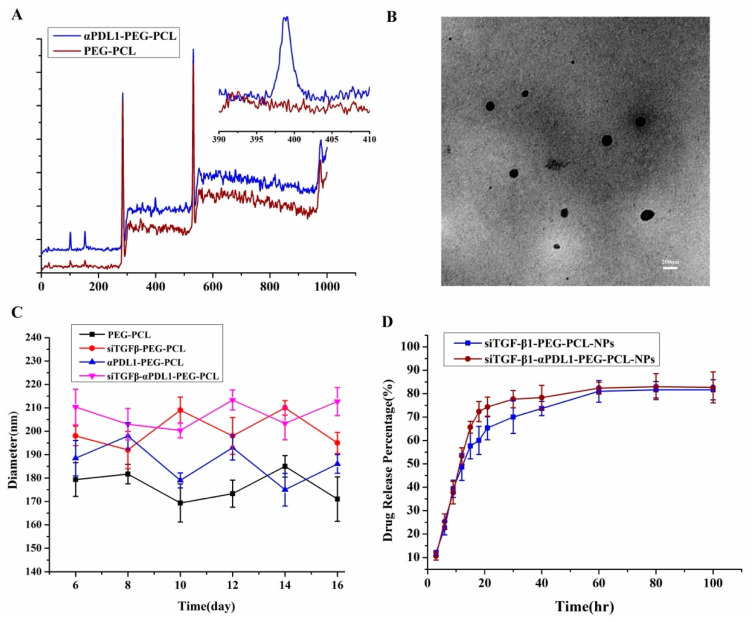
Characterization of siTGF-β1-αPDL1-PEG-PCL-NPs. (**A**) Representative XPS spectra and N 1s peaks (insets) of αPDL1-PEG-PCL NPs before (lower panel) and after (upper panel) αPDL1 conjugation. (**B**) Morphology of siTGF-β1- αPDL1-PEG-PCL-NPs by TEM. Scale bar, 200 nm. (**C**) Stability study of the produced NPs. The diameters of the NPs were determined by DLS, and data are mean ± SD. (**D**) In vitro release of siTGF-β1 from αPDL1-PEG-PCL-NPs and PEG-PCL-NPs. Data are mean ± SD (XPS, X-ray photoelectron spectroscopy, siTGF-β1, Transforming Growth Factor-β1 siRNA, αPDL1, anti-programmed cell death-Ligand 1; TEM, transmission electron microscope; NPs, nanoparticles; DLS, Dynamic light scattering).

αPDL1-PEG-PCL synthesis was carried out as described above. Activated carboxyl groups in antibodies reacted with primary amino groups on PEG-PCL, linking antibodies on NPs. This antibody-NPs conjugation was verified by XPS. αPDL1-containing nitrogen atoms had more intense signals compared with PEG-PCL’s amino groups. Clearly distinguishable peaks depicting nitrogen (N 1s) suggested successful antibody conjugation in the matrix, although non-conjugated NPs equally presented reduced signals related to nitrogen in surface amino groups. Jointly, the above findings confirmed antibodies were successfully conjugated to the polymeric matrix (Figure 1A).

### 2.2. Ligand Surface Density

The association of antibody conjugates on NPs with PEG-PCL-to-αPDL1 ratio was examined. PEG-PCL/αPDL1 at 20%, 40%, 60%, and 80% (*w*/*w*) were used for αPDL1-PEG-PCL copolymer preparation, respectively. The final amounts of αPDL1 conjugated to NPs were 0.021 0.146, 0.274, and 0.256 mg/mg of NPs, respectively, on background subtraction based on 0% NPs (Table 1). Therefore, PEG-PCL/αPDL1 at 40% (*w*/*w*) was chosen for PDL1-PEG-PCL copolymer synthesis in subsequent assays.

### 2.3. Synthesis and Properties of TGF-β1 siRNA-Loaded NPs 

siTGF-β1-NPs and siTGF-β1-αPDL1-NPs were produced as described above. NPs without drug constituted controls. NPs averagely ranged between 179.7 and 210.5 nm in size, facilitating their enrichment in tumor tissues, with elevated permeability and retention (EPR) [23]. Zeta potential values were negative, ranging between −8.74 mV and −9.83 mV, and polydispersity ranged between 0.183 and 0.281 (Table 1). TEM (Figure 1B) demonstrated siTGF-β1-αPDL1-NPs were spherical, with averagely 200 nm in diameter. The DLCs of siTGF-β1 in siTGF-β1-NPs and siTGF-β1-αPDL1-NPs were 0.12 ± 0.012% and 0.14 ± 0.009%, respectively. The EE values of TGF-β1 siRNA in siTGF-β1-NPs and siTGF-β1-αPDL1-NPs were 50.22 ± 4.3% and 58.3 ± 4.4%. The αPDL1 contents of siTGF-β1-NP and siTGF-β1-αPDL1-TNP were 5.83 ± 0.21% and 4.92 ± 0.55%, respectively (Table 2). This αPDL1-NP formulation yielded a αPDL1-to-TGF-β1 siRNA mass ratio of 32.6:1.

### 2.4. Structural Stability, TGF-β1 siRNA Protection and Release from NPs In Vitro

siTGF-β1-αPDL1-NPs and siTGF-β1-NPs had stable sizes for >14 days (Figure 1C). Serum stability was assessed for determining the protective abilities of αPDL1-NPs and NPs toward siRNA. Free TGF-β1 siRNA, siTGF-β1-PEG-PCL and siTGF-β1-αPDL1-NPs underwent incubation in FBS at 37 °C for various times. The results revealed PEG-PCL and αPDL1-NPs protected TGF-β1 siRNA from nucleases for up to 12 h (Figure 1D). Meanwhile, free TGF-β1 siRNA was totally degraded within 6 h. These findings indicated PEG-PCL and αPDL1-PEG-PCL NPs had high stability in serum. TGF-β1 siRNA release occurred rapidly from αPDL1-NPs and NPs for the initial 10 h, with sustained release in the subsequent 96 h.

### 2.5. Cell Uptake 

**Figure 2:**
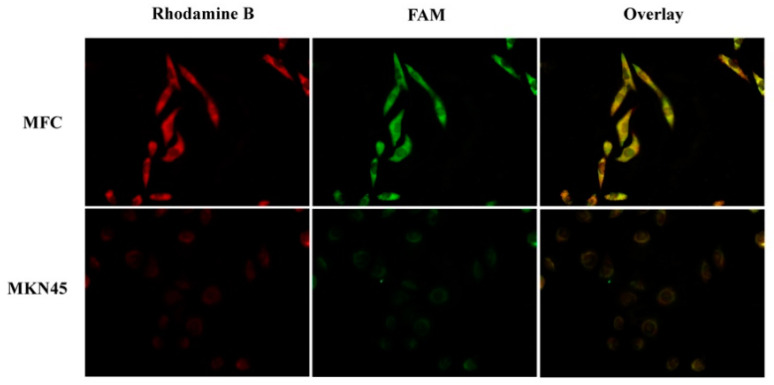
Fluorescent images of siTGF-β1-αPDL1-PEG-PCL-NPs in MFC and NUGC4 cells. NPs labeled with the fluorescent dye rhodamine B (red) were assessed for cellular uptake. Transfected cells were observed via siTGF-β1 labeled with FAM (green). Scale bar, 100 μm (NPs, nanoparticles; siTGF-β1, Transforming Growth Factor-β1 siRNA; αPDL1, anti-programmed cell death-Ligand 1).

The cell uptake of αPDL1-PEG-PCL NPs was examined on the basis of αPDL1 amounts. Rhodamine-labeled FAM-TGF-β1 siRNA-αPDL1-NPs and FAM-TGF-β1siRNA-NPs were used for tracking the NPs. Fluorescent green signals of FAM-TGF-β1 siRNA and red signals of rhodamine B-labeled NPs co-localized in the cytosol (Figure 2), indicating TGF-β1siRNA entry into the cytoplasm together with NPs. The MKN45 cell line was used as a negative control for comparing cells not expressing PDL1. The IOD values of Rhodamine B and FAM were starkly elevated in the αPDL1-PEG-PCL group of MFC cells in comparison with that of MKN45 cells (*p* < 0.001) (Table 3).

### 2.6. Establishment of MFC cells Expressing TGF-β and In Vivo Efficacy Study

**Figure 3:**
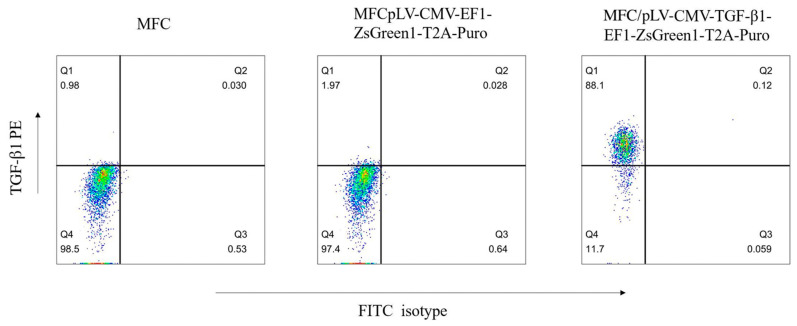
Flow cytometry analysis of TGF-β1 expression in parental and transduced cells. Cells (1 × 10^6^) were incubated with PE-anti-mouse TGF-β1 for 30 min, and then analyzed by flow cytometry with the Expo32 ADC software. TGF-β1 was expressed in >88% MFC/TGF-β1 cells, while little expression was found in other cells.

MFC cells underwent transfection with pLV-CMV-EF1-ZsGreen1-T2A-Puro and pLV-CMV-TGF-β1-EF1-ZsGreen1-T2A-Puro, and MFC/TGF-β1 cells were further analyzed. Flow cytometry was carried out to assess TGF-β1 amounts in both parent and transfected cells (Figure 3). In TGF-β1 + MFC tumor-engrafted mice, Blank NPs and siTGF-β1-NPs showed no tumor growth suppression. Treatment with αPDL1 and αPDL1-PEG-PCL had similar and higher antitumor effects compared with saline (*p* < 0.01). Tumors administered αPDL1 + siTGF-β1-NPs and siTGF-β-αPDL1-PEG-PCL were markedly inhibited in comparison with the αPDL1 and αPDL1-PEG-PCL groups (*p* < 0.01). Moreover, the antitumor effect of siTGF-β1-αPDL1-NPs was more pronounced than that of αPDL1 + siTGF-β1-NPs starting from 2 weeks after treatment initiation. The superiority of the antitumor effect of siTGF-β-αPDL1-PEG-PCL kept increasing with time (*p* < 0.01). SiTGF-β1-αPDL1-NPs totally suppressed tumor growth, more pronouncedly compared with other test articles (*p* < 0.01). Tumors administered siTGF-β-αPDL1-PEG-PCL were the smallest among the totality of groups (*p* < 0.01). Of note, siTGF-β1-NPs and saline had similar antitumor effects (*p* > 0.05; Figure 3), likely because TGF-β1 mainly regulates cancer cell proliferation. 

### 2.7. TGF-β1 Silencing and Immunological Phenotypes Changed by TGF-β1 siRNA

**Figure 4:**
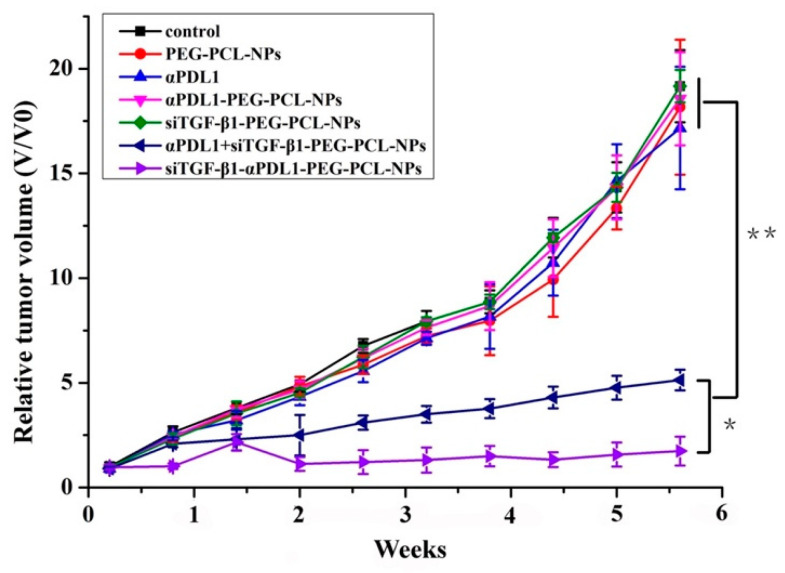
Antitumor efficacies of siTGF-β1-αPDL1-PEG-PCL-NPs for different treatments upon relative tumor volumes in TGF-β1 + MFC cell-engrafted mice (* *p* < 0.05, ** *p* < 0.01).

**Figure 5:**
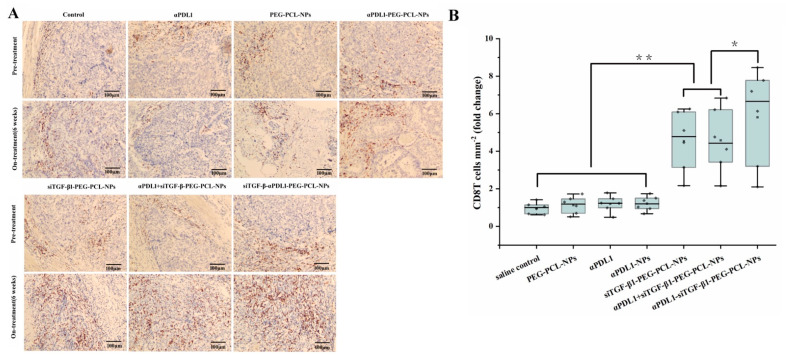
(**A**) Representative images of immunohistochemically stained CD8+ T cells in the center of TGF-β1 + MFC 615 mouse tumors before and after different treatments (from up to down, saline control, PEG-PCL-NPs (NPs), αPDL1, αPDL1-NPs, siTGF-β1-PEG-PCL-NPs αPDL1 + siTGF-β1-PEG-PCL-NPs and αPDL1-siTGF-β1-PEG-PCL-NPs). (**B**) Quantification of mean cell densities (cells/mm^2^) for immunohistochemically stained CD8+ T cells. Images represent three independent experiments, N = 6 mice for all groups. (TGF-β1, Transforming Growth Factor-β1; * *p* < 0.05, ** *p* < 0.01).

On the basis of the above observations that the “immune-excluded” phenotype of gastric cancer is characterized by high expression of TGF-β1, the efficacy of siTGF-β1-αPDL1-PEG-PCL-NPs against GC could be enhanced by down-regulating suppressive cytokines, such as TGF-β1, within the tumor microenvironment, therefore increasing CD8+ T cell infiltration in the tumor parenchyma and converting the immune-exclude phenotype into the immune-inflamed phenotype. To test this hypothesis, tumor-specific silencing of TGF-β1 was achieved by systemic delivery of siTGF-β1 with αPDL1-PEG-PCL-NPs and PEG-PCL-NPs. q-PCR data confirmed an efficient down-regulation of TGF-β1 in the tumor tissue (Figure 4). In addition, the TGF-β1 + MFC model and saline control groups were similar, while αPDL1, PEG-PCL-NPs and αPDL1-PEG-PCL-NPs exhibited the immune-excluded phenotype via tumor tissue detection from MFC mice by immunohistochemistry (Figure 5A). The siTGF-β1-PEG-PCL, αPDL1 + siTGF-β1-PEG-PCL and siTGF-β1-αPDL1-PEG-PCL groups showed significantly enhanced CD8+ T cell infiltration into the tumor, converting the immune-excluded phenotype into the immune-inflamed phenotype (Figure 5A). Moreover, T cell distribution was markedly altered by the above therapies; the number of infiltrated CD8+ T cells was higher in the tumor tissue after treatment with siTGF-β1-αPDL1-PEG-PCL-NPs compared with the siTGF-β1-PEG-PCL and αPDL1 + siTGF-β1-PEG-PCL groups (Figure 5B). Jointly, the above findings indicated TGF-β1 suppression increased the potential of anti-PD-L1 in enhancing antitumor immunity, which results in optimal T cell positioning and subsequent tumor regression. 

### 2.8. Correlation between TGF-β1 and Cancer-Immune Phenotypes in Gastric Cancer Tissues

**Figure 6:**
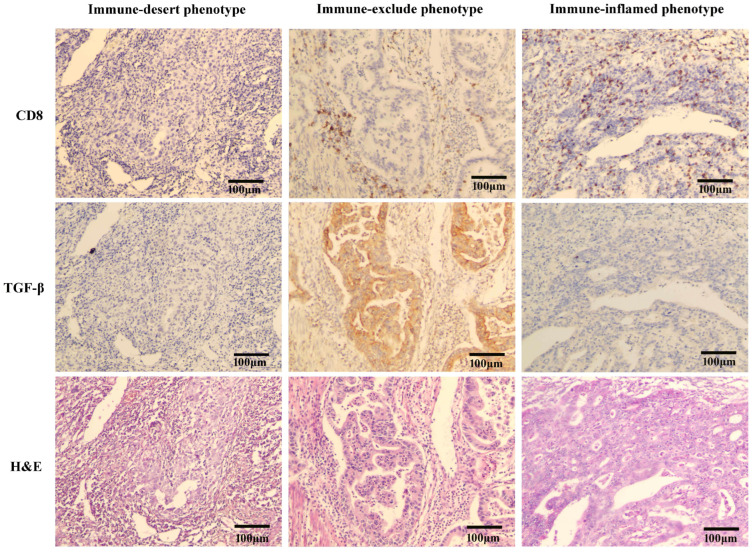
Representative images of CD8+ T cells and TGF-β1 in the center of human gastric cancer tissues. Immune-inflamed phenotype, CD8+ T cells were mainly located in the tumor and the paracancerous stroma; immune-excluded, CD8+ T cells are mainly located in the paracancerous stroma; immune-desert, CD8+ T cells were not overtly seen in the tumor or paracarcinoma (TGF-β1, Transforming Growth Factor-β1).

The expression of CD8 was detected by immunohistochemical staining to obtain the distribution of CD8+ T cells in gastric cancer. Immunohistochemistry revealed the rate of immune-inflamed phenotypes (CD8+ T cells mainly located in tumor cells and paracancerous stroma, Figure 9) in gastric cancer tissues was 33.3%. The rate of immune-excluded phenotype (CD8+ T cells mainly located in the paracancerous stroma; Figure 6) in gastric cancer tissues was 51.2%. The rate of immune-desert phenotype (CD8+ T cells with no obvious expression in tumor cells and paracarcinoma; Figure 6) in gastric cancer tissues was 15.5%. Immune-excluded tumors might correspond to particular chemokine state, vascular factors/barriers, or stromal-based suppression. Immunohistochemistry showed that the positive expression rates of TGF-β1 in these three phenotypes (immune-inflamed, immune-excluded and immune-desert) were 61.1%, 88.4%, and 69.2%, respectively, and TGF-β1 was mainly located in the cytoplasm of gastric cancer cells (Figure 6). TGF-β1 expression was significantly positively correlated with the immune-excluded phenotype (*p* = 0.0009, Table 4).

## 3. Discussion

RNA interference (RNAi) represents a pathway that suppresses gene expression after transcription, which is induced by double-stranded RNAs (dsRNAs) such as endogenous microRNAs (miRNAs) and synthetic short interfering RNAs (siRNAs) [24]. Indeed, siRNAs can efficiently and specifically silence almost all genes, even those routinely regarded as ‘undruggable’ [25]. RNAi has great therapeutic prospects, and siRNA-based drugs are being developed for several pathologies, from viral infections [26] to cancers [27] Therefore, in this study, TGF-β1 siRNA was applied to down-regulate the expression of TGF-β1. However, proper application of siRNA-based drugs requires safe and efficient delivery tools. Indeed, siRNA has low stability in circulation, could be immunogenic and cannot readily enter cells [28]. Besides the hurdle of exerting therapeutic effects, regulating the highly pleiotropic cytokine TGF-β1 may cause substantial systemic toxicity, since TGF-β1 plays important roles in maintaining immune cell homeostasis [29]. Systemic TGF-β1 signaling suppression might induce autoimmunity, as demonstrated in TGF-β1 knockout mice that developed multifocal inflammation and autoimmunity [30]. Humans not expressing TGF-β1 show elevated risk of autoimmune pathologies, including rheumatoid arthritis and systemic lupus erythematosus [31]. Thus, TGF-β1 inhibitors administered systemically should be thoroughly assessed for the balance between efficacy and safety. The efficacy of TGF-β1 inhibitors could be increased by targeting them to the TME, which may unfold their full potential while reducing systemic adverse effects. 

Antibodies blocking PD-1/PD-L1 signaling trigger substantial and lasting responses in multiple cancers clinically. However, such responses are not found in all cases. Identifying factors that determine therapeutic response and resistance is important for outcome improvement and the development of novel treatment approaches. Response to PD-L1 drugs in clinical metastatic urothelial cancer is correlated with the CD8+ T-effector cell phenotype. Meanwhile, lack of response is related to a signature of TGF-β1 signaling effectors in fibroblasts, especially in individuals with tumors with CD8+ T cells excluded from the tumor parenchyma and instead located in the peritumoral stroma [17]. Consistently, we found that gastric cancer samples with highly expressed TGF-β1 had an immuno-excluded phenotype, both in TGF-β1 + MFC cell-engrafted mice (Figure 5A) or clinical gastric cancer specimens (Figure 6). Given this background, we have now extended our approach to combination therapy of ICI and TGF-β1 knockdown using TGF-β1 siRNA.

**Figure 7:**
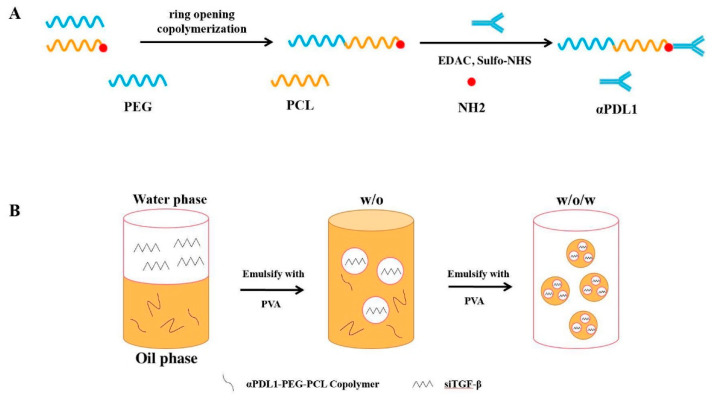
(**A**) Schematic illustration of the fabrication of αPDL1-conjugated PEG-PCL NPs. The NPs comprise a PCL core, a hydrophilic and stealth PEG shell on the surface of the core and a αPDL1 ligand coating layer. (**B**) Preparation of siTGF-β1-αPDL1-PEG-PCL-NPs (αPDL1, anti-programmed cell death-Ligand 1; NPs, nanoparticles; siTGF-β1, Transforming Growth Factor-β1 siRNA).

An important hurdle in exploiting the great potential of siRNA-based drugs is the lack of tools that can safely and effectively deliver them. Indeed, structural alterations and/or delivery tools are needed to transport siRNAs to the target sites with limited side effects. Various materials are currently being examined for improving in vivo delivery, e.g., polymers, lipids, peptides, antibodies, and aptamers [32]. Some delivery tools reduce siRNA degradation and promote immune evasion via encapsulation in NPs. NPs can be used for passively or actively targeting tumors [33]. Passive targeting involves the EPR effect [34]. In active targeting, NPs are decorated with targeting ligands, including antibodies and peptides, with specificity to receptors overexpressed at the tumor site. Most siRNA delivery systems enter the cell via endocytosis. Multiple systems attempt to improve cell uptake via incorporation of targeting ligands specifically binding to receptors on target cells for enhancing receptor-induced endocytosis [35]. We used tumor cell-specific NPs linked to antibodies, e.g., αPDL1. In a previous report, our team linked antibodies (e.g., trastuzumab) to NP surface for targeted delivery of anti-miR21 [22]. In this work, PEG-PCL NPs were linked to αPDL1 for targeted delivery of TGF-β1 siRNA (Figure 7A). αPDL1-PEG-PCL NP preparation used one step carbodiimide coupling with EDAc and Sulfo-NHS as described above (Figure 7B). Unbound amino groups on NPs reacted with the ligand. Elevated amounts of amino groups in PEG-PCL resulted in higher efficiency of covalent conjugation. At a PEG-PCL-to-αPDL1 ratio of 25%, maximal binding occurred. The best formulation was next assessed for TGF-β1 siRNA delivery into MFC cells. A αPDL1-to-PEG-PCL ratio of 3:1 (*w*/*w*) resulted in effective conjugation (Table 1). NPs produced by double-emulsion solvent evaporation approximated 200 nm in size and had negative zeta potential values, promoting endocytosis-induced cell uptake [23]. As demonstrated above, the particle sizes of PEG-PCL NPs, αPDL1-PEG-PCL-NPs and TGF-β1 siRNA-αPDL1-PEG-PCL-NPs were stable for more than 14 days (Figure 1C). This promoted the loading and protection of TGF-β1 siRNA (Figure 1D), which may also occur in circulation and in the TME. Besides being stable in aqueous milieux, sustained TGF-β1 siRNA release in vitro indicates it effectively diffuses from NPs for efficient silencing (Figure 1D).

**Figure 8:**
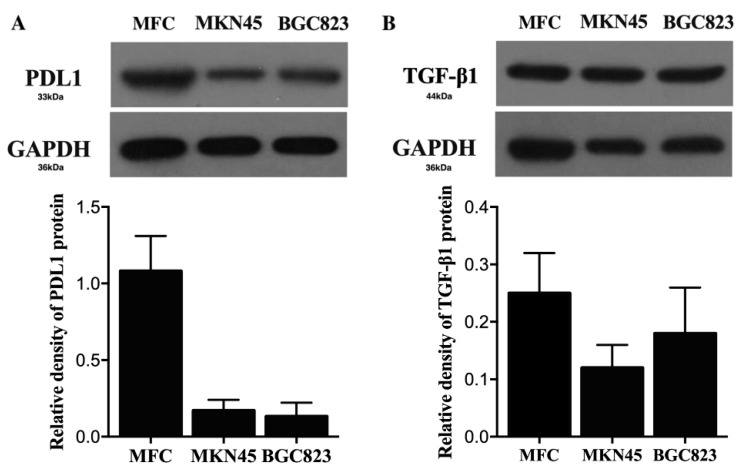
Detection of PDL1 and TGF-β1 expression levels. (**A**) Western blot analysis and protein quantification of PDL1 expression in MFC, MKN45 and BGC823 cells. (**B**) Western blot analysis and protein quantification of TGF-β1 expression in MFC, MKN45 and BGC823 cells (αPDL1, anti-programmed cell death-Ligand 1; TGF-β1, Transforming Growth Factor-β1).

Nanocarriers and nontarget cells can nonspecifically interact, limiting treatment effects and causing deleterious effects [27]. Using fluorescent TGF-β1 siRNA and FAM-TGF-β1 siRNA (green) and rhodamine B-labeled αPDL1-PEG-PCL NPs (red), cellular uptake of siRNA was examined under a fluorescence microscope. FAM-TGF-β1 siRNA was detected in the cytosol in MFC cells post-transfection. Next, targeted uptake was examined, using MKN45 cells showing low PD-L1 expression (Figure 8A) as a control. Compared with MFC cells overexpressing PD-L1 (Figure 8B), reduced uptake levels of FAM-TGF-β1 siRNA-αPDL1-NPs and Rhodamine B in MKN45 cells further verified αPDL1-NP entry into MFC cells occurs via αPDL1 antibody-mediated endocytosis. This finding demonstrated formulating drugs with a PDL1-targeting ligand improves cell uptake and enhances transfection, enabling interactions with overexpressed antigens on the cell surface. Furthermore, the nano-formulation encompassing αPDL1 linked to NPs had increased effects due to active entry into tumor cells that express αPDL1.

**Figure 9:**
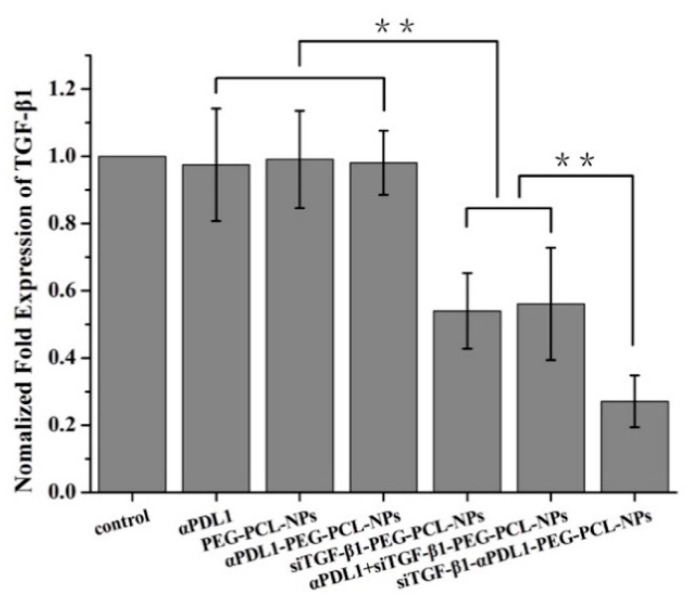
Expression of TGF-β1 in tumor tissue samples from TGF-β1 + MFC mice after different treatments (from up to down: saline control, PEG-PCL-NPs (NPs), αPDL1, αPDL1-NPs, siTGF-β1-PEG-PCL-NPs, αPDL1 + siTGF-β1-PEG-PCL-NPs and αPDL1-siTGF-β1-PEG-PCL -NPs) after 6 weeks. The expression of TGF-β1 was normalized to the U6 small nuclear RNA gene (U6 snRNA) control. Compared with the saline control group, the expression levels of TGF-β1 in the PEG-PCL-NPs (NPs), αPDL1, αPDL1-NPs, siTGF-β1-PEG-PCL-NPs, αPDL1 + siTGF-β1-PEG-PCL-NPs and αPDL1-siTGF-β1- PEG-PCL-NPs groups were 0.98-fold (*p* > 0.05), 0.99-fold, 0.98-fold, 0.52-fold, 0.56-fold and 0.27-fold (siTGF-β1, Transforming Growth Factor-β1 siRNA; NPs, nanoparticles; αPDL1, anti-programmed cell death-Ligand 1; ** *p* < 0.01).

Mariathasan et al. found that cancer cells may secrete cytokines that surround them. The resulting microenvironment traps T cells, preventing them from reaching the cancer cells to perform their functions, i.e., promoting the immune-excluded phenotype [17]. In addition, high expression of TGF-β1 was found in the latter phenotype. In a mouse model recapitulating this phenotype, co-treatment with TGF-β1-blockers and anti-PD-L1 antibodies helped T-cells penetrate into the tumor center, provoking substantial antitumor immunity and causing tumor shrinkage [17,18]. Compared with the above therapeutics, siRNA design and production could be much more efficient and faster [36]. As shown above, αPDL1-NPs linked to PEG-PCL and free αPDL1 had comparable antitumor effects in mice implanted MFC cells, indicating the retention of antibody activity even in produced NPs. Additionally, siTGF-β1-αPDL1-NPs totally inhibited tumor growth, with much higher efficacy compared with the other test articles. The siTGF-β1-αPDL1-NPs had elevated tumor regression rate in comparison with other products (Figure 4A). The above findings suggest TGF-β1 siRNA may constitute a sensitizer of αPDL1 in the case of entrapment in siTGF-β1-αPDL1-NPs, indicating that the enhanced anti-tumor effect of siTGF-β1-αPDL1-NPs in TGF-β1 + MFC mouse is mediated by targeted delivery. The cell interaction and enhanced transfection markedly reduced TGF-β1 mRNA amounts in comparison with PEG-PCL NPs in TGF-β1 + MFC cells (Figure 9). Therefore, αPDL1 exerts its effect as a targeting ligand and improves TGF-β1 transfection in PDL1+ cells. αPDL1-conjugated NPs had elevated and more direct effects in comparison with PEG-PCL NPs alone in TGF-β1 + MFC cell-implanted mice.

In conclusion, PD-L1 blockage and TGF-β down-regulation as monotherapies showed minimal effects in TGF-β1 + MFC cell-implanted mice. The group administered TGF-β1 siRNA-αPDL1-NPs targeting PD-L1 with TGF-β1 silencing had significantly decreased tumor burden (Figure 4). To further clarify the anti-tumor mechanism, qPCR showed TGF-β1 siRNA-αPDL1-NPs led to a significant decrease of TGF-β1 expression levels (Figure 9), which increased the number of CD8+ T cells in the tumor center and converted the immune-excluded phenotype to the inflamed phenotype (Figure 5), causing robust antitumor immunity and reducing tumor burden. This finding corroborated Mariathasan et al. who assessed urothelial cancer. In addition, the above data demonstrated TGF-β up-regulation in the gastric TME is a major mechanism of immune evasion that induces T cell exclusion and prevents the occurrence of the CD8+ T phenotype. Immunotherapeutic drugs targeting TGF-β signaling might consequently be broadly applied in clinic for advanced GC.

The ATTRACTION-2 (ClinicalTrials.gov: NCT02267343) study that led to PD-1 approval in many countries (Japan, Korea, Taiwan and Switzerland) for cases of unresectable advanced and/or recurrent gastric cancer after progression following chemotherapy demonstrated ICB treatment (median OS of 5.3 months) results in superior overall survival (OS) in comparison with placebo (median OS of 4.1 months) [37]. Unfortunately, despite the considerable success of the above study, only a subset of patients could benefit from nivolumab (overall response rate [ORR] = 11.9%) [37]. Moreover, some cases progressed again after clinical remission [38]. Hence, it is an urgent need to increase the efficacy of ICB treatment in GC. The majority of solid tumors in humans exclusively show the immune-inflamed, immune-excluded, or immune-desert phenotype [39]. Previously, melanoma studies indicated inflamed tumors show highest response to checkpoint inhibitors, but whether and how the tumor immune phenotype affects treatment response in GC remains undefined. In the current gastric cancer cohort, we found that the immune-inflamed, immune-excluded, and immune-desert phenotypes in gastric cancer represented 33.3%, 51.2%, and 15.5%, respectively (Figure 6, Table 4). The rate of immune-excluded phenotype was the highest among the three tumor phenotypes, which is consistent with Mariathasan et al.’s study of urothelial cancer [17]. In addition, there was a significant correlation between the rate of the immune-excluded phenotype and the expression of TGF-β1 (Table 4). Most of the gastric cancer patients with high expression of TGF-β1 were of the immune-excluded phenotype. Combining these clinical data and the above in vivo findings, this study suggests the potential of ICB therapy combined with anti-TGF-β therapy for gastric cancer, which may also bring new hope for ICB therapy in gastric cancer.

Studies assessing targeted PEG-PCL-based delivery in combination with ligands for siRNA delivery in gastric cancer are scarce. Here, PEG-PCL NPs targeting PD-L1 were synthesized for delivering TGF-β1 siRNA to gastric cancer. The siTGF-β1-αPDL1-NPs had favorable physicochemical features and were selectively taken up by targeted cells. αPDL1-NPs with TGF-β1 siRNA significantly knocked down TGF-β1 and enhanced CD8+ T cell infiltration from stroma to tumor center after initiation of therapy. The above data indicate siRNA therapies could be potentially combined with ICI in GC. This work is an initial step in developing TGF-β1 siRNA and nanotechnology-based therapeutics for GC. Additionally, in the 84 examined clinical specimens, immune-exempt gastric cancers accounted for the majority (51.2%). The ability of αPDL1-PEG-PCL NPs to transfect siRNAs and to channel therapeutic antibodies to tumor cells could help develop a multifaceted approach for GC treatment. The efficacy of siTGF-β1-αPDL1-NPs in targeting gastric cancer will be further investigated.

## 4. Materials and Methods

### 4.1. PEG-PCL and αPDL1-PEG-PCL Copolymer Production

PEG-PCL copolymer production was based on a previous report [40]. Then, αPDL1 (10F.9G2; BioXCell, Lebanon, NH, USA) conjugates were developed as previously proposed [19]. In brief, dry PEG-PCL NPs underwent incubation with αPDL1 in borate buffer containing EDAc (N-(3-Dimethylaminopropyl)-N0-ethylcarbodiimide hydrochloride) and Sulfo-NHS (N-Hydroxysulfosuccinimide, Shanghai, China), overnight at ambient. The samples were centrifuged, and the resulting pellet was resuspended with ultrapure water after washing, for subsequent assays. The supernatant was assessed for antibody amounts at 595 nm on a microplate plate Reader (Synergy HT, BioTek, Winooski, VT, USA). The antibody amounts on NPs were obtained as initial levels minus supernatant amounts.

### 4.2. Preparation of TGF-β1siRNA Loaded NPs

TGF-β1 siRNA (GenePharm, Changsha, China)-containing PEG-PCL and PDL1-PEG-PCL NPs, respectively, were prepared as described previously [40]. Briefly, TGF-β1 siRNA or FAM-TGF-β1 siRNA was mixed with spermidine at a polyamine nitrogen to polynucleotide phosphate (N/P) ratio of 10:1. TGF-β1 siRNA- or FAM-TGF-β1 siRNA-containing polyamine complexes were prepared at ambient for 15 min with shaking. The mixture was emulsified with a copolymer (3 mg) in dichloromethane, and 5% polyvinyl alcohol (PVA) was added for achieving a double emulsion (Figure 7). NPs obtained by centrifugation underwent washing and lyophilization. Antibody levels were assessed as described above.

### 4.3. Surface Chemistry

αPDL1 on NPs were assessed by X-ray photoelectron spectroscopy (XPS). The NP surface was also assessed for elements’ specific binding energy (eV), which ranged between 0 and 1000 eV (pass energy, 80 eV) under fixed transmission. Nitrogen was examined with the fine mode at 0.5 eV. Data processing used a specific XPS software.

### 4.4. NP Properties and Drug Loading Efficiency

NP size and polydispersity were determined by dynamic light scattering (DLS) (Brookhaven Instruments, Holtsville, NY, USA). A Zetaplus (Brookhaven Instruments) was used for zeta potential measurements. Sample storage was performed at 37 °C in PBS. NP morphology was determined by transmission electron microscopy (TEM) on a JEM-100S (JEOL, Tokyo, Japan). One drop of NP suspension placed on a copper grid was covered with nitrocellulose membrane, followed by air-drying at ambient and negative staining with phosphotungstic sodium solution (1% *w*/*v*). Finally, the encapsulation efficiency (EE) and drug loading content (DLC) of TGF-β1 siRNA were determined as proposed previously [40]. DLC and EE were derived as follows:

$$\mathrm{DLC}\%=\frac{\mathrm{Weight}\;\mathrm{of}\;\mathrm{the}\;\mathrm{drug}\;\mathrm{in}\;\mathrm{nanoparticles}}{\mathrm{Weight}\;\mathrm{of}\;\mathrm{the}\;\mathrm{drug-loaded}\;\mathrm{nanoparticles}}\;\times \;100\%\;$$

$$\mathrm{EE}\%=\frac{\mathrm{Weight}\;\mathrm{of}\;\mathrm{the}\;\mathrm{drug}\;\mathrm{in}\;\mathrm{nanoparticles}}{\mathrm{Weight}\;\mathrm{of}\;\mathrm{the}\;\mathrm{feeding}\;\mathrm{drug}}\;\times \;100\%\;$$

### 4.5. Structural Stability and TGF-β1 siRNA Protection and Release from NPs In Vitro

NPs, αPDL1-NPs, TGF-β1 siRNA-NPs (siTGF-β1-NPs) and TGF-β1 siRNA-αPDL1-NPs (siTGF-β1-αPDL1-NPs) were kept at 37 °C in PBS. Their sizes were assessed for 16 days to determine structural stability. A serum stability test indicated αPDL1-NPs could escape nuclease degradation. In brief, siTGF-β1-NPs, siTGF-β -αPDL1-NPs and free TGF-β1 siRNA underwent incubation with 50% fetal bovine serum (FBS) at 37 °C for several times. Specimens were loaded onto an agarose gel (1.5% *w*/*v*) with ethidium bromide. Then, the release levels of siTGF-β1-αPDL1-NPs and siTGF-β1-NPs were assessed after addition of giving NP amounts to RNAse- and DNAse-free PBS and dialysis with a MWCO of 3500 Da. TGF-β1 siRNA amounts were assessed by the QuantiT^TM^, PicoGreen^TM^ assay.

### 4.6. Cell Culture

A mouse gastric cancer cell line mouse forestomach carcinoma (MFC) cells, and human gastric cancer cell line MKN45 and BGC823 cells, were provided by the Chinese Academy of Medical Science Shanghai, underwent culture in RPMI 1640 containing 10% FBS, 100 U/mL penicillin and 100 mg/L streptomycin at 37 °C in a 5% CO_2_ incubator. Totally 1 × 10^6^ cells were plated per culture dish. PD-L1 and TGF-β1 levels were examined by immunoblot. PD-L1 was highly expressed by MFC cells, while being lowly expressed by MKN45 and BGC823 cells (Figure 8A). Meanwhile, TGF-β1 was lowly expressed in these three types of cells (Figure 8B). Our preliminary experiments also indicated evidently reduced TGF-β mRNA in MKN45 and BGC823 cells (data not shown). Therefore, MFC and MKN45 cells were selected for further study.

### 4.7. Cellular Uptake Studies

Rhodamine B-labeled particles reacted with hydroxyl groups in NPs and αPDL1-NPs [30]. The labeled NPs underwent loading with FAM-conjugated siTGF-β1 (FAM-siTGF-β1), and fluorescence was used for tracking siTGF-β1 and NPs in MFC and MKN45 cells. Cells seeded in 6-well plates at a density of 2 × 10^5^/well underwent overnight incubation. Then, fixed amounts of FAM-siTGF-β1-loaded Rhodamine B-labeled NPs and αPDL1-NPs were supplemented for another 2-h incubation. After fixation with 4% formalin for 30 min at ambient and washing, fluorescence was measured under an Axio Scope A1 fluorescence microscope (Zeiss, Jena, Germany). NPs and αPDL1-NPs groups were assessed for color change with ImageJ. Fluorescence was quantitated based on integrated optical density (IOD). Rhodamine B and FAM were assessed for IOD by carrying out triplicate assays under similar conditions.

### 4.8. Cell Infection Procedure

As described above, we first measured PD-L1 and TGF-β1 amounts in MFC cells by Western blot. The results revealed PD-L1 had strong positive signals, while TGF-β1 was barely detected that is PD-L1 + TGF-β-MFC. Therefore, we engineered MFC cells expressing TGF-β1 through stable transfection. MFC cells were infected by pLV-CMV-TGF-β16-EF1-ZsGreen1-T2A-Puro (Uptbio, Changsha, China) following routine protocols. The cells were incubated in 96-well plates at a density of 4 × 10^3^/well for overnight. In addition, the lentivirus mixtures were added to the cell plates for a further incubation for 24 h. Subsequently, after cell fluorescence was observed under a fluorescence microscope, the lentivirus was further cultured with MFC cells for 48 h. Finally, PD-L1 + TGF-β1 + MFC cells were harvested for further in vivo assays.

### 4.9. Flow Cytometry

To assess TGF-β1 amounts, 10^6^ cells underwent incubation with PE-conjugated anti-mouse TGF-β1 antibodies (BioXCell, Lebanon, NH, USA) for 30 min at ambient, followed by filtration through a 500-mesh copper screen prior to flow cytometry analysis on an Epics-XLII (Beckman Coulter, Indianapolis, IN, USA). PE-rat IgG (BioXCell, Lebanon, NH, USA) was used as an isotype control.

### 4.10. Mouse Experiments

Specific pathogen-free (SPF) mice (Mice strain: 615; H-2Kk; 6–8 weeks old, 20–25 g) were provided by Tianjin Institute of Hematology, The Chinese Academy of Medical Science. Totally 50 animals were used, with equal amounts of males (N = 25) and females (N = 25). Experiments involving animals had approval from the institutional Animal Welfare and Ethics Committee. Exponentially growing TGF-β1 + MFC cells were resuspended at 5 × 10^7^/mL, and 0.2 mL of this suspension was administered in the right armpit. After 10 days, MFC cell indurations reached 3–7 mm in diameter, and were administered to mice by subcutaneous inoculation. At tumor volumes of 50–100 mm^3^, TGF-β1 + MFC tumor-harboring mice were assigned to seven groups (six animals/group with 3 male mice and 3 female mice in each group), and administered saline (control group), PEG-PCL-NPs, siTGF-β1-PEG-PCL-NPs, αPDL1 antibodies (10F.9G2, BioXCell, Lebanon, NH, USA; 5 mg/kg), αPDL1-PEG-PCL-NPs (5 mg/kg αPDL1 eq), αPDL1 (5 mg/kg) + siTGF-β-PEG-PCL-NPs, and siTGF-β-αPDL1-PEG-PCL-NPs, (5 mg/kg αPDL1 eq), once every two weeks for 6 weeks via intraperitoneal injection, respectively. Tumors were measured every 4 days with calipers. Relative tumor volume was determined as follows:

$$\mathrm{relative}\;\mathrm{tumor}\;\mathrm{volume}=\frac{V}{\;V0}\;\times \;100\%\;$$

where V and V0 are absolute tumor volume and mean group tumor volume at Day 1, respectively.

When tumors were >2000 mm^3^, euthanasia was performed. Tumor samples were collected for further analysis.

### 4.11. Immunohistochemistry

Tumor tissues from the seven mouse groups were embedded and sectioned. For immunohistochemical studies, formalin-fixed sections underwent successive incubations with anti-CD8 primary (2 h at 37 °C) and secondary (1 h at ambient temperatures) antibodies′-diaminobenzidine chromogen (DAB) was used for development, followed by hematoxylin counterstaining.

### 4.12. Real-Time Quantitative RT-PCR (qPCR) for TGF-β1 Detection in Tumor Tissue Specimens

Total RNA isolation used TRIzol reagent (Invitrogen, Waltham, MA, USA) as directed by the manufacturer. Reverse transcription (based on 2 µg total RNA) employed a First Strand cDNA Synthesis kit (Promega, Madison, WI, USA). TGF-β1 mRNA amounts were assessed in tumor tissue specimens by qPCR with SYBR-Green (Stratagene, La Jolla, CA, USA) on a StepOnePlus Real-Time PCR System (Applied Biosystems; Waltham, MA, USA). Amplification was carried out for 40 cycles of 95 °C for 30 s and 60 °C for 1 min. The 2^−ΔΔCt^ method [41] was applied for quantitation, with glyceraldehyde-3-phosphate dehydrogenase (GAPDH) as a reference gene. The used primers are listed in Table 5.

### 4.13. Clinical Research

In total, 84 patients who underwent gastrectomy in the Affiliated Lianyungang Hospital of Xu Zhou Medical University from 2017 to 2019 were retrospectively enrolled in this study. The paraffin-embedded tissue samples and clinicopathologic features of these patients were collected. The immunohistochemical method was used to detect the expression of CD8 and to assess the distribution of CD8+ T cells in GC. TGF-β1 was also detected by immunohistochemistry to clarify the relationship between immunophenotypes. Formalin-fixed paraffin sections underwent successive incubations with anti-CD8 and anti-TGF-β1 primary (R&D; 2 h at 37 °C) and secondary (1 h at ambient temperatures) antibodies. DAB was used for development, followed by hematoxylin counterstaining. At the same time, hematoxylin and eosin (H&E) stained sections were used as controls to distinguish tumor cells, gastric stroma, and normal tissues, to determine the immunophenotype. The trial had approval from the institutional Ethics Committee, and signed informed consent was obtained from all patients.

## 5. Conclusions

The produced αPDL1-PEG-PCL NPs efficiently reduced the amounts of TGF-β1 mRNA in MFC cells, converting the immune microenvironment of MFC tumors engrafted mice from the “immune-excluded phenotype” to the “immune-inflamed phenotype”. PDL1-harboring gastric cancer had increased susceptibility to αPDL1. The value of this drug-controlled release system targeting the tumor microenvironment in immune checkpoint therapy of gastric cancer would provide a scientific basis for clinically applying nucleic acid drugs.

## 6. Declarations

### Ethics Approval and Consent to Participate

Experiments involving animals had approval from the institutional Animal Welfare and Ethics Committee. In addition, the human study had approval from the institutional Ethics Committee, and signed informed consent was obtained from all patients.

## Figures and Tables

**Table 1:** αPDL1 contents of αPDL1-PEG-PCL nanoparticles with various PEG-PCL amounts used in the nanoprecipitation process.

PEG-PCL to-αPDL1 Ratio (% *w*/*w*)	αPDL1 Content (% *w*/*w*) ^a^
20	2.1 ± 0.06
40	14.6 ± 0.15
60	27.4 ± 0.26
80	25.6 ± 0.19

^a^ SD of mean trastuzumab content obtained from three measurements.

**Table 2:** Mean particle size and drug loading efficiency in the four types of nanoparticles.

Nanoparticles	Diameters (nm) ^a^	Polydispersity ^a^	Zeta Potential (mV) ^a^	siTGF-β1 DLC (%) ^b^ siTGF-β1 EE (%) ^c^	αPDL1 Content (% *w*/*w*)
PEG-PCL-NPs	179.7 ± 5.6	0.183 ± 0.059	−8.77 ± 1.24	-	-
siTGF-β1-PEG–PCL-NPs	201.8 ± 3.7	0.207 ± 0.09	−8.96 ± 1.02	0.12 ± 0.012 50.22 ± 4.3%	-
αPDL1-PEG-PCL-NPs	188.9 ± 6.2	0.199 ± 0.063	−8.74 ± 1.07		5.83 ± 0.21%
siTGF-β1-αPDL1-PEG-PCL-NPs	210.5 ± 8.7	0.281 ± 0.075	−9.83 ± 1.33	0.14 ± 0.009 58.3± 4.4%.	4.92 ± 0.55%

^a^ SD value of mean particle size obtained from three measurements. ^b^ DLC, drug loading content. ^c^ EE, encapsulation efficiency.

**Table 3:** Integrated optical density (IOD) of Rhodamine B-αPDL1-PEG-PCL NPs and FAM-siTGF-β-αPDL1-PEG-PCL NPs in MKN45 and MFC cells.

Variable (IOD) ^a^	MKN45 ^b^	MFC ^b^	*p*
Rhodamine B (αPDL1-PEG-PCL-NPs)	54,311 ± 451.1	961,312 ± 593.6	<0.001 *
FAM (αPDL1-PEG-PCL-NPs)	42,113 ± 550.3	741,295 ± 491.2	<0.001 *

^a^ IOD, integrated optical density. ^b^ SD of mean IOD obtained from three measurements. The ImageJ software was used for analysis.

**Table 4:** Correlation between TGF-β and cancer-immune phenotypes in gastric cancer tissues (n).

Immune Phenotypes	Number (%)	TGF-β				*p*	
		−	+	++	+++		
immune-inflamed	28 (33.3)	11	4	9	4	0.2098 *p* > 0.05	0.0009 *p* < 0.05
immune-excluded	43 (51.2)	5	3	14	21	0.0434 *p* < 0.05	
immune-desert	13 (15.5)	4	2	5	2	0.3709 *p* > 0.05	

**Table 5:** Oligonucleotide and primer sets for qRT-PCR.

Oligonucleotide Sequences for miRNA Modulation	
Primer used for q-pcr TGF-β1-siRNAs forward TGF-β1-siRNAs reverse TGF-β1 forward TGF-β1 reverse GAPDH forward GAPDH reverse	5′-GCAACAACGCCAUCUAUGATT-3′ 5′-GCAACAACGCCAUCUAUGATT-3′ 5′-CTAAGGCGAAAGCCCTCAAT-3 5′-TGCACCAC CAACTGCTTAGC-3 5′-TGCACCACCAACTGCTTAGC-3 5′-GGCATGGACTGTGGTCATGAG-3

## Data Availability

Data is contained within the article.
